# Localized chilling of crowns promotes floral bud differentiation in strawberry transplants in a closed transplant production system

**DOI:** 10.1093/aobpla/plaf004

**Published:** 2025-01-27

**Authors:** Jaewook Shin, Meiyan Cui, Hyein Lee, Byungkwan Lee, Jeesang Myung, Haeyoung Na, Changhoo Chun

**Affiliations:** Department of Agriculture, Forestry and Bioresources, Seoul National University, 1, Gwanak-ro, Gwanak-gu, Seoul 08826, Korea; Research Institute of Agriculture and Life Sciences, Seoul National University, 1, Gwanak-ro, Gwanak-gu, Seoul 08826, Korea; Department of Agriculture, Forestry and Bioresources, Seoul National University, 1, Gwanak-ro, Gwanak-gu, Seoul 08826, Korea; Department of Agriculture, Forestry and Bioresources, Seoul National University, 1, Gwanak-ro, Gwanak-gu, Seoul 08826, Korea; Department of Agriculture, Forestry and Bioresources, Seoul National University, 1, Gwanak-ro, Gwanak-gu, Seoul 08826, Korea; Major in Horticultural Science, Mokpo National University, 1666, Yeongsan-ro, Cheonggye-myeon, Muan 58554, Korea; Department of Agriculture, Forestry and Bioresources, Seoul National University, 1, Gwanak-ro, Gwanak-gu, Seoul 08826, Korea; Research Institute of Agriculture and Life Sciences, Seoul National University, 1, Gwanak-ro, Gwanak-gu, Seoul 08826, Korea

**Keywords:** chilled crown, crown temperature, floral bud differentiation, June-bearing strawberry, transplant production

## Abstract

A stable supply of transplants with floral buds is required to improve the initial yield of the June-bearing cultivars of strawberry (*Fragaria* × *ananassa* Duch.). A closed transplant production system (CTPS) enables year-round production to meet the demands for the year-round production of strawberries in plant factories. In this study, we evaluated the performance of a novel method involving the localized chilling of strawberry crowns using silicone tubes containing circulated chilled water at different temperatures (10, 15, or 20°C) at the nighttime and different chilling regimes (daytime, nighttime, or entire day) under high air temperature conditions in a CTPS in terms of floral bud differentiation. We observed that 4 weeks of localized chilling at 10 or 15^o^C during the nighttime under the air temperature of 25/20°C (photo-/dark periods) and a photoperiod of 10 h promoted floral bud differentiation, whereas 6 weeks of localized chilling under the same conditions inhibited differentiation. Moreover, 4 weeks of localized chilling at 5^o^C during the daytime or entire day under the elevated air temperatures of 28/21°C and an extended photoperiod of 14 h promoted floral bud differentiation, and 6 weeks of localized chilling during the entire day under the same conditions further promoted bud differentiation compared with that in the control. Plant growth was generally unaffected by the localized chilling of the crowns. The results indicate that to cope with the impacts of elevated air temperature and photoperiod conditions, the continuous localized chilling of crowns at 5^o^C during the entire day for 6 weeks must be used to achieve optimal bud differentiation. These findings suggest the effectiveness of the localized chilling of the crowns for floral bud differentiation in strawberry in CTPSs, without disrupting the high-air temperature and long-day conditions required for vegetative growth.

## Introduction

Strawberry (*Fragaria* × *ananassa* Duch.) production has increased drastically during the last decade and has expanded to 77 countries worldwide (Simpson, 2018). The June-bearing cultivars of *F*. × *ananassa* exhibit seasonal flowering and environmental sensitivity and undergo floral bud differentiation induced by low temperatures and short days (Darrow, 1936; Ito and Saito, 1962; Jonkers, 1965; Manakasem and Goodwin, 1998). The production of June-bearing strawberry cultivars in greenhouses is restricted to a few months a year, including winter, in northern temperate regions because of the environmental requirements for floral bud differentiation. To meet the recent demands for the year-round production of strawberries (Yanagi, 2017; Stein, 2021), June-bearing production in large-scale plant factories has been evaluated (Iwao *et al.*, 2021; Le *et al.*, 2021). A stable year-round transplant production and supply must be ensured to improve the efficiency and productivity of large-scale production in plant factories.

A closed transplant production system (CTPS) is a thermally insulated structure wherein disease-free transplants can be produced in a controlled environment (Park *et al.*, 2020), which can be utilized for year-round transplant production (Kozai *et al.*, 2000; Kim *et al.*, 2010; Chun *et al.* 2012). Plug transplant production using unrooted runner tips in CTPSs exhibits the advantages of high propagation rate, uniform quality, and efficient utilization of space and resources (Durner *et al.*, 2002). CTPSs adopt the autotropic transplant production method, wherein the smallest-sized runner plants that can survive independently are used as the next-generation propagules (Chun *et al.* 2012; Park *et al.*, 2020; Lee *et al.*, 2023). The simulated cumulative number of transplants per year from a CTPS with a cultivation area of 72 m^2^ exceeds 55 000 (Park *et al.*, 2020). Environmental conditions in CTPSs must be modified to improve vegetative growth and support a highly efficient transplant production system.

Early and sufficient floral bud differentiation in strawberry transplants ensures an early yield (Jun *et al.*, 2013). Although environmental conditions in plant factories are controlled for ensuring stable floral bud differentiation, insufficient floral bud differentiation in transplants can delay initial fruit production. During transplant production in CTPSs, high air temperatures and long days promote vegetative growth, limiting natural floral bud differentiation. The modification of environmental conditions during the final phase of each transplant production cycle to initiate floral bud differentiation places a burden on the facility and hinders the growth of other transplants in previous growth stages. The development of localized temperature control methods to accelerate floral bud differentiation without affecting the environment of the entire system is required to improve the efficiency of CTPSs.

Various chilling methods have been developed to promote floral bud differentiation in strawberry transplants under high air temperatures in greenhouses (Mukai and Ogura, 1988; Ikeda *et al.*, 2007; Yamazaki *et al.*, 2007). Typical methods include shifting transplants to cold rooms (Jun *et al.*, 2013; Li *et al.*, 2021). However, this process is labor-intensive (Choi *et al.*, 2023), and prolonged cold storage in the dark affects initial growth after transplanting (Yoshida and Nishimoto, 2020). The supply of light during chilling can improve vegetative and reproductive growth (Li *et al.*, 2021), highlighting the importance of a suitable light environment during chilling. A few studies have suggested the localized chilling of transplants under light, such as cooling the nutrient solution at night (Mukai and Ogura, 1988) and cooling the root zone during short days (Mizuno *et al.*, 2022). Mizuno *et al.* (2022) inferred that low soil temperatures act as cold stimuli on shoot apical meristem, promoting flowering. Most previous studies have focused on the indirect regulation of air and root zone temperatures, and only limited studies have addressed localized temperature control in plants.

Plant meristem temperatures has rarely been quantified, using air temperature as a proxy (Savvides *et al.*, 2013). However, plant meristems in plants are sensitive to ambient temperatures (Peacock, 1975; Metzger, 1988; Fortin and Poff, 1990), which affects floral bud development (Song *et al.*, 2013; Lin *et al.*, 2019). The strawberry plant has a crown, which is a short and thick stem containing a shoot apical meristem (Poling, 2012; Dan *et al.*, 2015), which may be involved in temperature-induced floral bud differentiation. The flowering is suggested to be a sucrose-mediated cytokinin-induced response (Darnell *et al.*, 2003). Differences in day and night temperatures result in sucrose accumulation, which increases cytokinin, thereby inducing flowering (Tanino and Wang, 2008). Despite the importance of the crown as a carbohydrates source during reproductive development (Macías-Rodríguez *et al.*, 2002), little information is available on variations in crown temperature and the relationship between crown temperature and floral bud differentiation. Hidaka *et al.* (2017) applied cooling pipes made of polyvinyl chloride near the crowns and controlled the temperature of water circulating in these pipes in a greenhouse. They found earlier than normal flowering on the first inflorescence as water temperatures decreased under high air temperatures (30/27^o^C).

In this study, we devised a novel system to control the crown temperature of strawberry transplants in a CTPS. To promote floral bud differentiation and maintain air temperature and photoperiod within the suitable ranges for strong vegetative growth, we investigated the physiological and developmental responses of strawberry transplants to the localized chilling of the crowns using this novel methods involving silicone tubes. The relationships between crown temperatures, diurnal chilling timings, and floral bud differentiation were examined.

## Materials and methods

### Plant materials and cultivation conditions

June-bearing strawberry plants (*F*. × *ananassa* Duch. cv. Seolhyang) were propagated in a CTPS under cool white light-emitting diodes (TTCC20365E01E9; Namyung Co., Seoul, Korea) under a photosynthetic photon flux density of 160 μmol m^−2^ s^−1^ and a photoperiod of 16 h, as described previously with a slight modification (Lee *et al.*, 2023). Runner tips with unfolded bracts were produced and fixed on 32-cell plug trays (150 mL/cell) filled with a commercial growing medium (Baroker; Seoul Bio Co. Ltd., Eumseong, Korea) containing essential microelements. The plants were subirrigated for 45 min d^−1^ with the modified Yamazaki solution for strawberry (NO_3_–N, 5.0 me L^−1^; NH_4_–N, 0.5 me L^−1^; PO_4_–P, 1.5 me L^−1^; K, 3.0 me L^−1^; Ca, 2.0 me L^−1^; Mg, 1.0 me L^−1^; and S, 1.0 me L^−1^; Yamazaki, 1984) at an electrical conductivity of 1.2 dS m^−1^ and a pH of 6.0.

### Chilled crown treatments

**Fig. 1.**
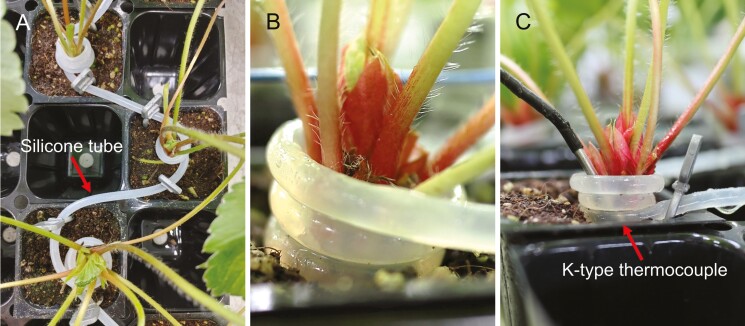
The arrangement of a silicone tube for chilling of the crowns (A), a strawberry crown coiled with a silicone tube (B), and a K-type thermocouple for measuring crown temperature (C) in the chilling treatments.

Plants with four unfolded leaves and a crown diameter of 8 mm were used for the treatments. For localized chilling treatments, silicone tubes (⌀ 5 mm) were coiled 2.5 times around the base of each crown such that they did not disrupt leaf development (Fig. 1A and B). Water was chilled using a chilling machine (DA-500B; Daeil Co., Busan, Korea). The plants were either subjected to crown chilling temperatures at 10, 15, and 20^o^C or to no chilling treatment. All plants were exposed to ambient temperatures (25/20^o^C). For the chilling treatments, the chilled water was circulated in the silicone tubes during the nighttime for 6 weeks under a photoperiod of 10 h and relative humidity of 65/95% in the photo-/dark periods.

The localized chilling treatments under diurnal chilling regimes were evaluated independently of the localized chilling treatments at 10, 15, and 20^o^C during the nighttime. In the diurnal chilling treatments, the crowns were chilled at 5^o^C during the daytime, nighttime, or entire day, under the elevated air temperatures of 28/21^o^C. The control plants for these treatments were exposed to the elevated air temperatures of 28/21^o^C with no chilling. The photoperiod for these treatments was extended to 14 h, as a previous study reported that June-bearing cultivars require a day length of no longer than 14 h for floral initiation (Darrow, 1936).

### Measurements of environmental variables and plant growth characteristics

#### Crown temperatures and environmental conditions

The crown temperatures were measured using K-type thermocouples connected to a data logger (UA11-K; Radionode Co., Yongin, Korea) using the Tapaculo Lite (v. 3.49) software. The thermocouples were positioned between each chilling tube and the crown to ensure close contact with the crowns (Fig. 1C). The crown temperature data were collected over a period of 40 days and then averaged. The air temperature and relative humidity data were recorded using a thermorecorder (TR-72wb; T&D Co., Nagano, Japan).

#### Floral bud development

**Fig. 2.**
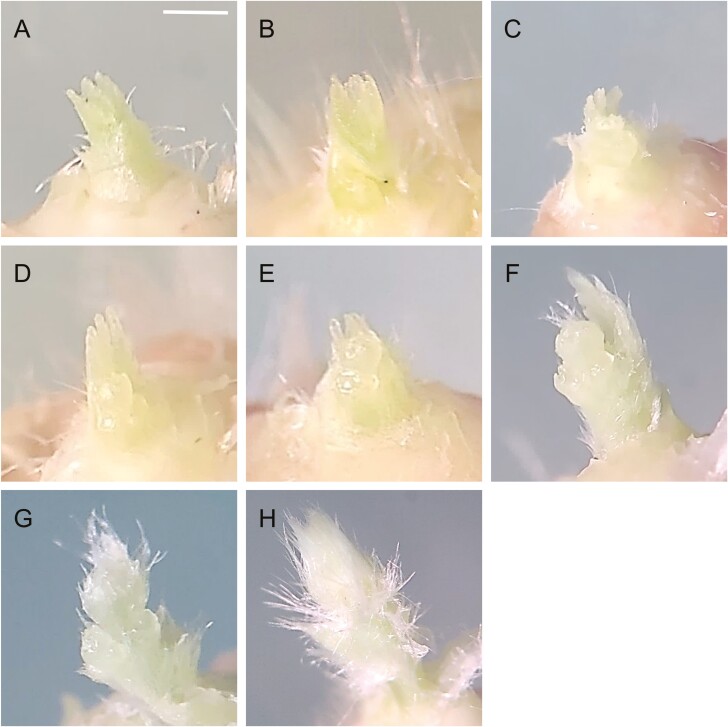
Microphotograph of the floral bud differentiation stages based on the apical meristems in the chilled crowns of strawberry transplants. The microscopic examination classified floral bud differentiation: 1, vegetative apex (A); 2, primary flower primordium initiation (B); 3, sepal development (C); 4, petal initiation (D); 5, sepal and petal development (E); 6, stamen development (F); 7, epidermal hair development (G); and 8, enclosed primary primordium (H). Bar = 1 mm.

Following 4 and 6 weeks of treatments, six strawberry transplants were randomly selected from 16 transplants for each treatment category. All unfolded leaves were removed, and the folded leaves enclosing the meristem were removed using a knife. To examine floral buds, apical meristems were observed under a dissecting microscope (KSZ-1B; Samwon Scientific, Seoul, Korea). Floral bud differentiation stages were classified into eight stages as described by Jahn and Dana (1970), with slight modifications: 1, vegetative apex; 2, primary flower primordium initiation; 3, sepal development; 4, petal initiation; 5, sepal and petal development; 6, stamen development; 7, epidermal hair development; and 8, enclosed primary primordium (Fig. 2) [see Supporting Information Table S1].

#### Growth characteristics

Crown diameters, dry weights of leaf, crown, and root tissues, and leaf area were measured in six plants per treatment category after 4 and 6 weeks of treatments [see Supporting Information—Tables S2 and S3]. For the diurnal chilling treatments, the growth parameters of runner plants, including their number and dry weights, were measured [see Supporting Information—Tables S4 and S5]. The crown diameter was measured immediately above the root using digital calipers (Mitutoyo Co., Kawasaki, Japan). The dry weights were measured after drying the plant tissues at 80^o^C for a week. The total leaf area of each plant was measured using a leaf area meter (LI-3100; LI-COR, Lincoln, NE, USA).

### Statistical analyses

The experiments were performed in a completely randomized design, with 16 plants in each treatment category. All measurements were performed using a simple random sampling method. Statistical Analysis System (SAS) for Windows version 9.4 (SAS Institute Inc., Cary, NC, USA) was used to perform analysis of variance. If a significant treatment effect was observed, a comparison of the means was performed using Duncan’s multiple range test at *P* < .05. After 4 and 6 weeks of treatments, the floral bud development stages were ranked using Student’s *t*-test at *P* < .05.

## Results

### Crown temperatures

**Fig. 3.**
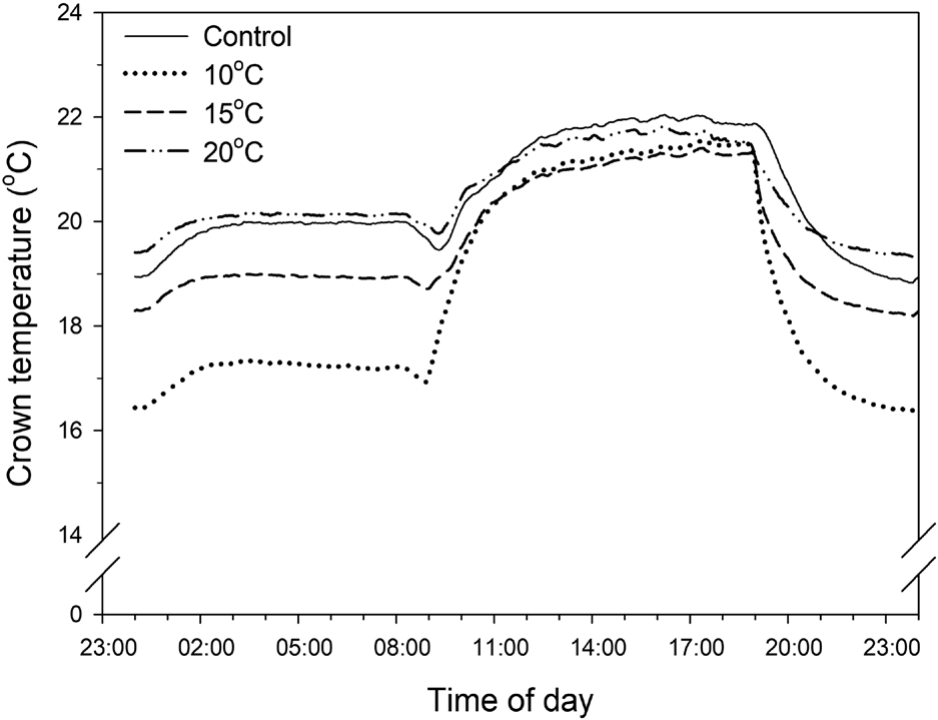
Daily crown temperatures of strawberry transplants as affected by chilling treatments. Control = 25/20^o^C. Chilling of the crowns was applied during the nighttime.

For all nighttime localized chilling treatments, the crown temperatures at night were the lowest with the chilling at 10^o^C (16.4–17.3^o^C), followed by those at 15^o^C (18.2–18.9^o^C), control temperatures (18.9–20.0^o^C), and 20^o^C (19.3–20.2^o^C) in that order (Fig. 3). Chilling at 20^o^C in the nighttime treatment resulted in higher crown temperatures at night than that in the respective control treatment, even though the crowns in control plants were exposed to ambient temperatures at night, which dropped to 20^o^C. The air temperature at night was sufficiently low; therefore, chilling at 20^o^C did not significantly decrease the crown temperature. The crown temperatures during the day in all nighttime treatments ranged from 20.5^o^C to 22.0^o^C, which was 3.5–5^o^C lower than the maximum air temperature (25^o^C).

**Fig. 4.**
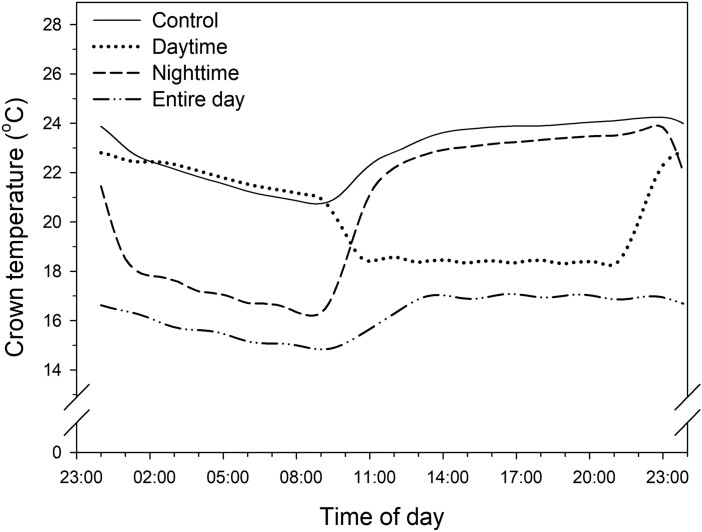
Daily crown temperatures of strawberry transplants as affected by the diurnal chilling treatments. Control = 28/21^o^C. The chilling temperature was 5^o^C.

For the diurnal localized chilling treatments, the chilling of the crowns at 5^o^C decreased the crown temperatures both during the day and night, even though the air temperatures were 28/21^o^C. The crown temperatures of the respective control plants ranged between 20.7^o^C to 24.2^o^C in a day (Fig. 4). Chilling during the daytime resulted in lower crown temperatures during the day than at night, ranging from 18.3–22.8^o^C. The crown temperatures decreased to 16.2^o^C with chilling during the nighttime. Chilling during the entire day maintained the crown temperature at 14.8–17.1^o^C in a day.

### Floral bud differentiation

**Fig. 5.**
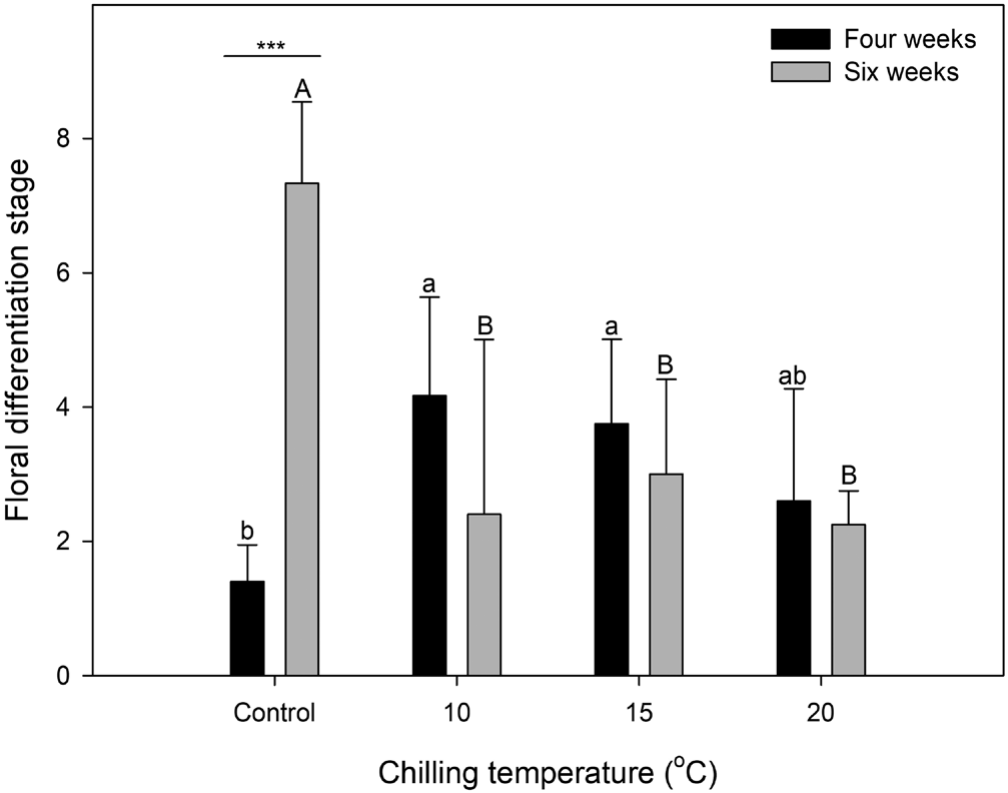
Floral bud differentiation in strawberry transplants as affected by 4 and 6 weeks of the chilling. Control = 25/20^o^C. Chilling of the crowns was applied during the nighttime. Different letters are considered significantly different according to Duncan’s multiple range test at *P* < 0.05. Asterisks indicate significant differences between the values in 4 and 6 weeks at each chilling treatment by Student’s *t*-test: *, *P* < 0.05; **, *P* < 0.01; ***, *P* < 0.001. The vertical error bars represent the standard error of the means (n = 6).

Chilling at 10 or 15^o^C for 4 weeks promoted floral bud differentiation compared with that in the respective control treatment, showing the floral bud differentiation stages of 4.2, 3.8, and 1.4, respectively (Fig. 5). However, after 6 weeks of treatment, the stages were relatively less advanced in the 10, 15, or 20^o^C treatment categories than in the respective control category. There was no significant difference in differentiation after 4 or 6 weeks of treatment across the chilling temperatures.

**Fig. 6.**
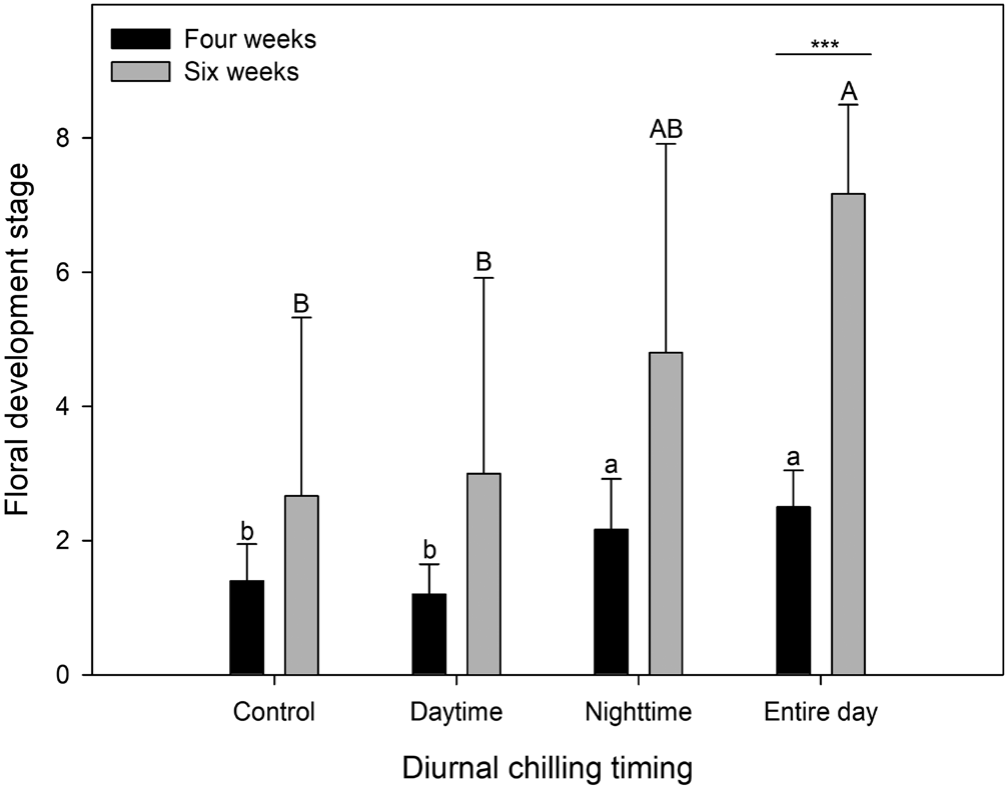
Floral bud differentiation in strawberry transplants as affected by 4 and 6 weeks of the diurnal chilling. Control = 28/21^o^C. The chilling temperature was 5^o^C. Different letters are considered significantly different according to Duncan’s multiple range test at *P* < 0.05. Asterisks indicate significant differences between the values in 4 and 6 weeks at each diurnal chilling treatment by Student’s *t*-test: *, *P* < 0.05; **, *P* < 0.01; ***, *P* < 0.001. The vertical error bars represent the standard error of the means (n = 6).

In the experiment with diurnal chilling at 5^o^C, the buds chilled during the nighttime or the entire day were at stages 2.5 and 2.2, respectively, after 4 weeks of treatment, whereas those chilled during the daytime and the control buds subjected to no chilling were at stages 1.2 and 1.4, respectively (Fig. 6). Differentiation after 6 weeks of treatment was highest with chilling during the entire day (stage 7.2), followed by that with chilling during the nighttime (Stage 4.8). Floral development was relatively more progressed after 6 weeks of chilling treatment during the entire day than that after 4 weeks of chilling treatment.

### Growth characteristics

Chilling of the crowns at different temperatures did not affect plant growth, except for higher dry leaf weights and greater leaf area after 6 weeks of chilling treatment at 20^o^C than that in the respective control category (Tables 1 and 2). After 4 weeks of diurnal chilling treatments at 5^o^C, the number of runner plants was greater in the respective control treatment and in the chilling treatment during the daytime than in the other treatment categories; however, the dry root weights were higher in chilling treatments during the nighttime or entire day than in the other treatment categories (Table 3). After 6 weeks of chilling treatments, the number and dry weights of runner plants were greater in the respective control treatment and in the chilling treatments during the daytime than in the other treatment categories (Table 4). However, chilling during the nighttime or the entire day resulted in higher dry crown weights than that in the chilling treatments during the daytime and the respective control treatment after 6 weeks of chilling treatments.

**Table 1.** Effect of chilling of the crown on crown diameter, dry leaf, crown, and root weights, and leaf area of strawberry plants after 4 weeks.

Chilling temperature (^o^C)^z^	Crown diameter(mm)	Dry weight (g/plant)			Leaf area (cm^2^/plant)
		Leaf	Crown	Root	
Control	9.6	1.9	0.2	0.3	463
10	10.8	2.4	0.3	0.4	527
15	11.0	1.6	0.2	0.3	398
20	10.8	2.1	0.2	0.4	408
*Significance* ^y^	ns	ns	ns	ns	ns

^z^Control = 25/20^o^C. Chilling of the crowns was applied during the nighttime.

^y^ns, not significant.

**Table 2.** Effect of chilling of the crown on crown diameter, dry leaf, crown, and root weights, and leaf area of strawberry plants after 6 weeks.

Chilling temperature (^o^C)^z^	Crown diameter (mm)	Dry weight (g/plant)			Leaf area (cm^2^/plant)
		Leaf	Crown	Root	
Control	11.8	2.8 ab^y^	0.3	0.5	621 ab
10	12.2	2.8 ab	0.5	0.7	501 bc
15	12.1	2.4 b	0.4	0.5	475 c
20	12.4	3.6 a	0.4	0.5	695 a
*Significance* ^x^	ns	^*^	ns	ns	^**^

^z^Control = 25/20^o^C. Chilling of the crowns was applied during the nighttime.

^y^Means within columns followed by different letters are significantly different by Duncan’s multiple range test at *P* < 0.05.

^x^Subscripts indicate:

^*^ *P* < 0.05;

^**^ *P* < 0.01;

^***^ *P* < 0.001; ns, not significant.

**Table 3.** Effect of chilling of the crown on crown diameter, number of runner plants, dry leaf, crown, root, and runner plant weights, and leaf area of strawberry plants after 4 weeks.

Diurnal chilling timing^z^	Crown diameter (mm)	No. of runner plants	Dry weight (g/plant)				Leaf area (cm^2^/plant)
			Leaf	Crown	Root	Runner plant	
Control	10.8	2.5 a^y^	2.1	0.2	0.2 b	0.6	400
Daytime	9.6	2.5 a	2.1	0.2	0.3 b	0.4	374
Nighttime	10.5	1.5 ab	2.4	0.3	0.4 a	0.2	423
Entire day	10.7	0.7 b	2.7	0.2	0.5 a	0.2	420
*Significance* ^x^	ns	^*^	ns	ns	^***^	ns	ns

^z^Control = 28/21^o^C. The chilling temperature was 5^o^C.

^y^Means within columns followed by different letters are significantly different by Duncan’s multiple range test at *P* < 0.05.

^x^Subscripts indicate:

^*^ *P* < 0.05;

^**^ *P* < 0.01;

^***^ *P* < 0.001; ns, not significant.

**Table 4.** Effect of chilling of the crown on crown diameter, number of runner plants, dry leaf, crown, root, and runner plant weights, and leaf area of strawberry plants after 6 weeks.

Diurnal chilling timing^z^	Crown diameter (mm)	No. of runner plants	Dry weight (g/plant)				Leaf area (cm^2^/plant)
			Leaf	Crown	Root	Runner plant	
Control	9.7	3.3 a^y^	2.4	0.3 bc	0.4	1.8 a	481 ab
Daytime	10.2	2.5 ab	1.8	0.2 c	0.4	1.2 b	354 bc
Nighttime	11.1	1.7 b	2.7	0.4 ab	0.7	0.5 c	495 a
Entire day	10.7	0.2 c	3.3	0.5 a	0.5	0.0 c	317 c
*Significance* ^x^	ns	^***^	ns	^*^	ns	^***^	^*^

^z^Control = 28/21^o^C. The chilling temperature was 5^o^C.

^y^Means within columns followed by different letters are significantly different by Duncan’s multiple range test at *P* < 0.05.

^x^Subscripts indicate:

^*^ *P* < 0.05;

^**^ *P* < 0.01;

^***^ *P* < 0.001; ns, not significant.

## Discussion

### Chilling temperatures and timings affected crown temperatures

Plant organ temperatures are affected by air temperatures, radiation, wind speed, and vapor pressure deficits (Novel, 2009). Savvides *et al.* (2013) suggested that plant meristem temperature is strongly related to bud structure and function. Strawberry crowns have a complex structure, including folded developing leaves and epidermal hairs, which can affect heat exchange between the crown and the environment.

The temperatures of the crown exposed to air and the crown in contact with the chilling tubes at 20^o^C were different at night in the different treatments, although the temperatures of the chilling tubes and air were the same. Our results are consistent with those reported in a previous study wherein the apical meristem temperatures of tomato and cucumber plants at night were lower than the air temperatures (Savvides *et al.*, 2013). The crown temperatures in the control plants were lower than the air temperatures at night.

Even though the water temperatures in all diurnal chilling treatments were the same at 5^o^C, chilling timings affected the crown temperatures in each of these treatment categories. These results indicate that the crowns remained exposed to air and the impact of air temperatures could not be avoided entirely.

### Localized chilling of crowns promoted floral bud differentiation without affecting the ambient environment of other plant parts

Floral bud differentiation in June-bearing strawberries is promoted by temperatures below 15^o^C, combined with short days (Jonkers, 1965; Bradford *et al.*, 2010). During the 4 weeks of chilling treatments at 10 or 15^o^C, the crown temperatures were lower than 19^o^C at night, which promoted floral bud differentiation. However, differentiation was inhibited after 6 weeks of chilling compared with that in the respective control treatment category. Lieten (2006) noted that excessive chilling delays floral bud differentiation and fruit production. According to Tehranifar *et al.* (1998), different strawberry cultivars require 2–4, 5–8, or more than 8 weeks of chilling for floral initiation. Li *et al.* (2021) have reported delayed floral bud differentiation in ‘Seolhyang’ transplants chilled at 8 or 15^o^C for 6 weeks in a cold room. Our results demonstrated that floral buds initiation occurred after 4 weeks of chilling of the crowns.

Our results revealed that chilling during the nighttime was crucial for floral bud differentiation. Chilling during the nighttime or the entire day promoted floral bud differentiation, whereas chilling during the daytime did not, likely because the average crown temperature daytime chilling treatment was higher than that during the nighttime or entire day chilling treatments. The air temperature is considered to impact differentiation (Darrow, 1936), and the increased air temperatures (28/21^o^C) during the diurnal chilling treatments may exert a negative impact on floral bud differentiation. Elevated air temperatures during floral differentiation can be counterbalanced by extending the chilling period; in this study, 6 weeks of localized chilling of the crowns during the entire day could effectively promote floral bud differentiation.

### Chilled crowns did not inhibit normal growth

We did not observe a significant reduction in shoot growth after the chilling treatments, indicating that localized chilling of the crowns using silicone tubes containing circulating chilled water did not cause physiological stress in treated plants and that the light conditions during the chilling treatments supported normal growth. Leaf growth and floral development directly contribute to crown growth during early development (Jahn and Dana, 1970). The increased dry crown weights after the chilling treatments during the nighttime or entire day may be associated with the promotion of floral bud development in the crowns. During floral bud development, temperatures lower than 2^o^C, can impair the viability of strawberry pollen (Cui *et al.*, 2023). Although the development of floral organs was not assessed, it was presumed that such negative effects of chilling treatments would be minimized, considering that the chilling temperature range was significantly different from extreme temperature conditions.

The vegetative and reproductive stages respond in opposite ways to photoperiod and air temperature conditions (Battey *et al.*, 1998; Konsin *et al.*, 2001), which interactively affect flower and runner plant development (Taylor, 2002). Greater chilling induces floral initiation and inhibits the development of runner plants, as runner growth occurs in the vegetative stage (Serce and Hancock, 2005; Tanino and Wang, 2008). The significantly decreased development of runner plants after the 6 weeks of chilling treatment during the nighttime or entire day indicates the clear transition to the reproductive stage.

## Conclusions

Based on the results obtained in this study, it can be inferred that the application of chilling treatments using the novel method for the localized chilling of strawberry crowns initiated floral bud development in strawberry transplants. Lower chilling temperatures were relatively more effective within 4 weeks of chilling. Moreover, 6 weeks of continuous chilling negatively impacted floral differentiation under the air temperatures of 25/20^o^C and a photoperiod of 10 h; however, the same duration was effective under the air temperatures of 28/21^o^C and a photoperiod of 14 h. The impact of air temperature on crown temperature could not be entirely avoided, indicating that extended chilling for 6 weeks throughout the entire day may be necessary to counterbalance elevated air temperatures. The localized chilling of the crowns enables efficient simultaneous production of strawberry transplants that require different environmental conditions in CTPSs.

## Supplementary Material

plaf004_suppl_Supplementary_Tables

## Data Availability

The data underlying this article are available in the article and in its online supplementary material.
