# Supplementary material for: Localized chilling of crowns promotes floral bud differentiation in strawberry transplants in a closed transplant production system
Source: AoB Plants. 2025 Jan 27;17(2):plaf004. doi: 10.1093/aobpla/plaf004 (PMC11799775; doi:10.1093/aobpla/plaf004)
Supplement: plaf004_suppl_Supplementary_Tables [file plaf004_suppl_supplementary_tables.pdf]

4) Supporting Information

Table S1. Raw data of floral bud differentiation stages in strawberry transplants as affected by 4 and 6 weeks of chilling of the crowns at different temperatures and diurnal regimes.

Treatment[z]	Floral bud differentiation stage
Control(25/20°C)_4W	2
Control(25/20°C)_4W	1
Control(25/20°C)_4W	1
Control(25/20°C)_4W	1
Control(25/20°C)_4W	2
Control(25/20°C)_6W	8
Control(25/20°C)_6W	7
Control(25/20°C)_6W	8
Control(25/20°C)_6W	8
Control(25/20°C)_6W	5
Control(25/20°C)_6W	8
10_4W	4
10_4W	3
10_4W	2
10_4W	5
10_4W	5
10_4W	6
10_6W	1
10_6W	1
10_6W	1
10_6W	2
10_6W	7
15_4W	5
15_4W	2
15_4W	4
15_4W	4
15_6W	3
15_6W	2
15_6W	5
15_6W	2
20_4W	1

20_4W	3
20_4W	1
20_4W	5
20_4W	3
20_6W	2
20_6W	2
20_6W	2
20_6W	3
Control(28/21°C)_4W	1
Control(28/21°C)_4W	1
Control(28/21°C)_4W	1
Control(28/21°C)_4W	2
Control(28/21°C)_4W	2
Control(28/21°C)_6W	1
Control(28/21°C)_6W	8
Control(28/21°C)_6W	2
Control(28/21°C)_6W	1
Control(28/21°C)_6W	2
Control(28/21°C)_6W	2
Daytime_4W	1
Daytime_4W	2
Daytime_4W	1
Daytime_4W	1
Daytime_4W	1
Daytime_6W	1
Daytime_6W	1
Daytime_6W	8
Daytime_6W	3
Daytime_6W	2
Nighttime_4W	1
Nighttime_4W	3
Nighttime_4W	3
Nighttime_4W	2
Nighttime_4W	2
Nighttime_4W	2
Nighttime_6W	7
Nighttime_6W	6
Nighttime_6W	8

Nighttime_6W	2
Nighttime_6W	1
Entireday_4W	3
Entireday_4W	2
Entireday_4W	3
Entireday_4W	3
Entireday_4W	2
Entireday_4W	2
Entireday_6W	8
Entireday_6W	5
Entireday_6W	8
Entireday_6W	8
Entireday_6W	6
Entireday_6W	8

[z]10, 15, and 20 refer to the chilling temperatures. Daytime, nighttime, and entireday refers to the diurnal chilling regimes. 4W and 6W indicate 4 and 6 weeks, respectively.

Table S2. Raw data of crown diameter, dry leaf, crown, and root weights, and leaf area of strawberry plants after 4 weeks of chilling of the crown.

Chilling temperature (°C)[z]	Crown diameter (mm)	Dry weight (g/plant)			Leaf area (cm^2/plant)
		Leaf	Crown	Root	
Control	9.02	1.295	0.112	0.237	353.65
Control	9.60	1.332	0.164	0.205	357.51
Control	10.08	2.74	0.277	0.412	605.26
Control	10.89	1.88	0.238	0.355	432.73
Control	9.88	3.04	0.297	0.356	757.33
Control	8.32	0.955	0.152	0.224	271.08
10	10.35	2.218	0.324	0.448	465.33
10	12.17	2.449	0.362	0.4	545.36
10	10.60	3.413	0.352	0.51	645.71
10	10.81	1.949	0.199	0.272	494.93
10	10.44	2.303	0.302	0.322	489.34
10	10.29	2.357	0.289	0.382	522.32
15	10.51	1.974	0.293	0.32	455.32
15	10.76	1.449	0.299	0.369	364.42
15	10.51	1.753	0.179	0.344	415.36
15	11.49	1.184	0.162	0.279	382.75
15	9.14	0.81	0.095	0.193	228.68
15	13.46	2.503	0.336	0.463	539.63
20	8.02	1.407	0.133	0.286	372.13
20	11.98	3.095	0.269	0.408	686.90
20	8.88	1.186	0.128	0.2	332.42
20	10.68	1.973	0.24	0.514	44.41
20	12.58	2.525	0.29	0.366	569.11
20	12.74	2.151	0.196	0.43	444.65

[z]Control = 25/20°C. Chilling of the crowns was applied during the nighttime.

Table S3. Raw data of crown diameter, dry leaf, crown, and root weights, and leaf area of strawberry plants after 6 weeks of chilling of the crown.

Chilling temperature (°C)[z]	Crown diameter (mm)	Dry weight (g/plant)			Leaf area (cm²/plant)
		Leaf	Crown	Root	
Control	10.79	2.945	0.473	0.469	579.57
Control	11.46	3.205	0.285	0.4	706.77
Control	13.13	3.057	0.18	0.498	674.67
Control	13.01	2.807	0.326	0.633	576.71
Control	10.15	2.358	0.196	0.277	582.94
Control	11.98	2.59	0.35	0.43	606.62
10	13.62	3.382	0.608	0.783	628.55
10	12.38	2.269	0.382	0.647	429.38
10	11.30	3.123	0.406	0.709	490.15
10	10.58	2.051	0.471	0.468	359.01
10	13.79	2.729	0.485	0.746	478.85
10	11.82	3.323	0.39	0.574	620.68
15	11.82	2.473	0.449	0.464	457.20
15	11.12	2.557	0.437	0.537	553.33
15	13.83	3.032	0.527	0.482	572.15
15	10.28	2.153	0.245	0.482	446.25
15	12.10	2.599	0.307	0.496	533.05
15	13.63	1.326	0.24	0.278	289.85
20	11.76	2.892	0.365	0.406	555.64
20	14.21	4.842	0.47	0.523	898.37
20	11.26	2.769	0.241	0.413	549.62
20	12.30	2.728	0.281	0.432	554.79
20	12.50	4.398	0.464	0.509	832.49
20	12.24	3.73	0.319	0.693	777.48

[z]Control = 25/20°C. Chilling of the crowns was applied during the nighttime.

Table S4. Raw data of crown diameter, number of runner plants, dry leaf, crown, root, and runner plant weights, and leaf area of strawberry plants after 4 weeks of chilling of the crown.

Diurnal chilling timing[z]	Crown diameter (mm)	No. of runner plants	Dry weight (g/plant)				Leaf area (cm^2/plant)
			Leaf	Crown	Root	Runner plant	
Control	10.9	2	2.294	0.231	0.217	0.561	505.87
Control	10.4	3	1.871	0.316	0.234	1.158	292.87
Control	9.8	1	1.690	0.280	0.307	0.148	270.29
Control	11.2	3	2.389	0.256	0.202	0.330	419.80
Control	11.2	3	1.927	0.184	0.234	0.674	408.08
Control	11.0	3	2.424	0.226	0.213	1.026	502.82
Daytime	9.6	4	1.759	0.164	0.179	0.661	366.57
Daytime	9.4	3	1.609	0.160	0.214	1.042	350.42
Daytime	9.5	3	2.952	0.301	0.283	0.158	418.76
Daytime	9.0	1	1.430	0.151	0.296	0.044	263.83
Daytime	10.9	3	2.944	0.293	0.355	0.430	519.21
Daytime	9.0	1	1.980	0.285	0.299	0.079	322.99
Nighttime	10.2	1	1.774	0.262	0.415	0.047	296.85
Nighttime	10.8	1	2.981	0.370	0.546	0.092	491.83
Nighttime	11.1	2	2.228	0.274	0.548	0.363	426.96
Nighttime	11.6	3	3.448	0.333	0.366	0.587	599.71
Nighttime	9.7	0	2.100	0.334	0.444	0.000	308.73
Nighttime	9.5	2	1.946	0.199	0.156	0.248	412.14
Entire day	9.2	0	1.800	0.205	0.327	0.000	357.45
Entire day	12.1	0	2.609	0.023	0.500	0.000	384.99
Entire day	10.5	3	3.054	0.229	0.504	0.671	580.92
Entire day	12.0	1	3.568	0.319	0.582	0.270	456.65
Entire day	10.4	0	1.825	0.267	0.566	0.000	317.67
Entire day	10.0	0	3.209	0.264	0.683	0.000	423.69

[z]Control = 28/21°C. The chilling temperature was 5°C.

Table S5. Raw data of crown diameter, number of runner plants, dry leaf, crown, root, and runner plant weights, and leaf area of strawberry plants after 6 weeks of chilling of the crown.

Diurnal chilling timing[z]	Crown diameter (mm)	No. of runner plants	Dry weight (g/plant)				Leaf area (cm^2/plant)
			Leaf	Crown	Root	Runner plant	
Control	10.03	3	3.077	0.239	0.559	1.779	558.51
Control	10.04	2	1.584	0.224	0.361	1.008	327.34
Control	9.37	4	2.531	0.215	0.338	1.505	540.86
Control	8.94	3	1.865	0.244	0.263	1.131	450.6
Control	11	4	3.137	0.383	0.498	2.708	603.34
Control	9.1	4	2.084	0.323	0.446	2.429	407.91
Daytime	9.87	4	1.803	0.234	0.408	1.692	332.15
Daytime	10.3	3	2.263	0.262	0.306	1.753	459.74
Daytime	9.18	1	1.351	0.148	0.4	0.133	182.02
Daytime	9.46	3	1.523	0.191	0.272	1.166	336.8
Daytime	10.8	3	1.943	0.221	0.256	1.168	432.34
Daytime	11.37	1	1.944	0.282	0.475	1.047	379.77
Nighttime	12.17	2	4.933	0.663	0.684	0.305	576.44
Nighttime	9.32	2	1.382	0.167	0.215	0.999	399.83
Nighttime	12.07	2	3.703	0.442	0.509	0.805	516.21
Nighttime	10.56	2	3.02	0.349	0.561	0.274	508.52
Nighttime	10.58	2	2.356	0.416	0.237	0.748	367.23
Nighttime	12.19	0	4.68	0.708	0.592	0	603.9
Entire day	9.65	0	2.052	0.353	0.344	0	249.04
Entire day	11.04	1	1.786	0.273	0.239	0.12	291.04
Entire day	10.77	0	3.133	0.454	0.626	0	344.47
Entire day	9.97	0	2.255	0.379	0.767	0	245.06
Entire day	12.92	0	5.213	0.764	1.332	0	557.06
Entire day	9.61	0	2.028	0.366	0.713	0	212.98

[z]Control = 28/21°C. The chilling temperature was 5°C.
